# First Year to Future Career: Women’s Engagement in Technical Participation Is Associated with Long-Term Retention

**DOI:** 10.3390/bs15020140

**Published:** 2025-01-27

**Authors:** Neil A. Lewis, Nicole Russell Pascual, Denise Sekaquaptewa, Lorelle A. Meadows

**Affiliations:** 1 Department of Communication, Cornell University, Ithaca, NY 14853, USA; 2 Department of Psychology, University of Michigan, Ann Arbor, MI 48109, USA; dsekaqua@umich.edu; 3 Department of Psychology and Human Factors, Michigan Technological University, Houghton, MI 49931, USA; lameadows@mtu.edu

**Keywords:** belonging, STEM retention, motivation, stereotypes, group dynamics

## Abstract

Societies realize the value of increasing the number of engineering and other STEM graduates, yet universities often struggle to enroll and retain STEM students, particularly women. To remedy this, many engineering programs have shifted their pedagogical approaches to include project-based learning in group settings. However, prior research on engineering teams revealed, for example, gender gaps in active participation, reflecting stereotypes of men as engineering experts and women as supporters. In the current study, we examined the long-term correlates of such gaps. Specifically, in a mixed-method study (behavioral observation, surveys, and longitudinal follow-up) we found gender differences in active technical participation during students’ first year in engineering project group presentations, such that men engaged in more active participation than women (*N* = 589). Longitudinal follow-ups in their final year revealed that first year technical participation was a predictor of feelings of belonging, and these feelings of belonging in turn predict retention in engineering majors and intentions to pursue graduate education in engineering. Together, these results suggest that the first year engineering team experience plays an important role in retaining students and highlight opportunities for early interventions.

## 1. First Year to Future Career: Women’s Engagement in Technical Participation Is Associated with Long-Term Retention

Increasing the proportion of students who obtain undergraduate degrees in Science, Technology, Engineering, and Mathematics (STEM) is important for the growth of the global economy, and thus multiple government agencies in the United States and elsewhere have set goals to increase undergraduate STEM participation (e.g., President’s Council of Advisors on Science and Technology, 2012; U.S. Department of Education, 2022). These goals are obstructed by several challenges, including under-enrollment and attrition among STEM students. Enrollment rates in the STEM fields are low, with only around 28% of U.S. students, for example, choosing to pursue a STEM major (Chen & Soldner, 2013). Of the students who enroll into STEM majors, many leave either due to changing majors or not completing their college degree (Chen & Soldner, 2013). For example, 40% of initially enrolled students leave engineering and 60% leave computing (Hill & Corbett, 2015). Graduation rates in STEM over time have remained relatively stable; for example, graduation rates in engineering have only increased from 6% in 1970–1971 to 7% in 2020–2021 (National Center for Education Statistics, 2022).

Some evidence suggests that these rates are similar for men and women within the field of engineering (Ohland et al., 2008; Orr et al., 2013). However, among some sub-disciplines, such as computer science and engineering, women are disproportionately underrepresented (Deitz & Henke, 2023; National Center for Science and Engineering Statistics, 2023; National Girls Collaborative Project, 2024). Global statistics highlight that this issue extends beyond the United States, as women are less likely to graduate in STEM fields in numerous countries (World Bank Group, 2020). In addition to being less likely to pursue STEM fields at an undergraduate level, women are less likely to remain within the engineering profession post-graduation (Frehill, 2012; Hunt, 2015). These trends are worrisome for two reasons. First, they undermine the goal of increasing the quantity of the STEM workforce. Second, given the body of research documenting the benefits of diversity in work teams (for review, see Page, 2007, 2019), lowered retention of women undermines the quality of said workforce.

Given these trends of STEM attrition and their implications for society, many universities have employed strategies to increase retention of underrepresented undergraduates in STEM fields, including developing living-learning communities (e.g., WiSTEM Living Learning Community; University of Texas, 2024), prescriptive academic programs (e.g., Meyerhoff Scholars Program; Maton et al., 2000) and academic assistance programs for students struggling with course content (e.g., Batz et al., 2015; Lewis & Yates, 2019). In addition, many engineering schools and colleges have shifted their pedagogical approaches to include project-based learning in group work settings. The goal of this shift is to increase student exposure to relevant and applied activities and development of technical skills, in concert with key professional skills such as teamwork and communication (Haidet et al., 2014; Nuñez-Thompson et al., 2023; Severiens & Schmidt, 2009; Vaz et al., 2013). With this growing exposure to group work settings early in the undergraduate engineering curriculum, students have opportunities to work closely with individuals from a variety of backgrounds and perspectives as they establish their personal and professional identities. These activities not only provide social benefits, but they also provide several advantages for student learning. Active participation in diverse teams promotes long-term retention of knowledge (Strobel & van Barneveld, 2009) and the development of higher-order cognitive skills (Michaelsen et al., 2014) and improves overall academic performance (Barkley et al., 2014; Haidet et al., 2014; Mckeachie et al., 1986).

In addition to gains in academic performance, another possible benefit of working in student groups is that working collaboratively in teams may lead students to feel a greater sense of belonging in the field (Love et al., 2014). By feelings of belonging, we mean students’ perceptions that they will be welcomed and accepted by their peers and other experts (e.g., the faculty) within their field (Freeman et al., 2007). In fields like engineering that employ project-based learning approaches, working on group projects provides students with the opportunity to develop skills that they can authentically apply in their disciplines both while in school and later in their careers (Thomas, 2000). Therefore, successful participation in group projects that develop these skills may lead students to perceive themselves, and to be perceived by others, as having the competence required to succeed in their field (e.g., Nuñez-Thompson et al., 2023). Since competence is one of the primary dimensions that people use to evaluate others (Fiske et al., 2002), being perceived as competent should increase the odds of being accepted by others in the field, and hence students’ feelings that they are a valued member of the discipline.

In the context of STEM learning environments, and engineering in particular, the technical aspects of group projects require the greatest technical expertise or experience. In other words, those who can successfully engage with technical content in engineering are perceived to be the most competent (Trevelyan, 2013). Specifically, students’ level of technical participation—activities that require use of technical, computational, and/or engineering design skills—can convey to others that they are knowledgeable about the critical aspects of engineering and can be counted on to take leading roles in their groups. Being perceived in this way is consequential, and, in turn, predicts students’ sense of belonging in their field. For example, researchers have found that, for engineering students, perceptions of technical competence are highly correlated with a sense of belonging in engineering (Wilson et al., 2010).

The association between technical competence and sense of belonging in engineering likely occurs because developing a perception of competence in engineering can help students develop a positive “possible self” as a successful engineer. Possible selves are elements of people’s self-concept that represent what they can become (Ruvolo & Markus, 1992), and research in this area suggests that these self-concepts motivate action in relevant social contexts (Luttenberger et al., 2019; Oyserman, 2015; Oyserman et al., 2017). That is, being in contexts or engaging in behaviors (such as technical participation) that make salient the idea that “people like me” can succeed in this domain is a powerful way to increase engagement and persistence (Aelenei et al., 2017; Lewis & Oyserman, 2016; Lewis & Sekaquaptewa, 2016; Luttenberger et al., 2019). This may be particularly powerful for people who routinely experience contexts in which an opposite narrative is the dominant one—people from groups whose abilities are negatively stereotyped in a domain, such as women (Aelenei et al., 2017; Lewis & Sekaquaptewa, 2016). Prior research has demonstrated that, for those who typically face such contexts, having opportunities to engage in tasks that allow them to demonstrate their competence is important for persistence in school and success in other domains of life (for review, see Oyserman, 2015).

On the other hand, when there are discrepancies between how people perceive themselves and how those in their environments treat them, that can undermine psychological well-being (Barnett et al., 2017; Mason et al., 2019). In other words, not having opportunities to demonstrate their competence can undermine the development of students’ productive possible selves. Applying these concepts to the engineering and broader STEM learning contexts, engaging with technical aspects of the curriculum may provide underrepresented students with opportunities to demonstrate their competence, which may ultimately lead to feeling more included in engineering spaces.

Feeling included in their learning environment is also important for students’ long-term success (G. M. Walton & Cohen, 2007, 2011). A large body of educational research shows that feeling included in school is positively associated with the capacity for building self-efficacy and the development of intrinsic motivation (Anderman & Anderman, 1999; Battistich et al., 1995; Goodenow, 1993). In the field of engineering, prior research has documented that confidence in skills plays a significant role in short-term persistence in engineering degree programs (Cech et al., 2011). However, for persistence into the field post-graduation, the dominant factor is sense of fit (belonging) within engineering culture and norms (Cech et al., 2011). Beyond academic goals, a sense of belonging also relates to students’ career aspirations (Meehan & Howells, 2019). Together, this research suggests that fostering a sense of belonging is key for long-term outcomes in engineering. Taking these findings into consideration, it is important to foster a sense of belonging, if students from diverse backgrounds are to persist in and beyond their academic pursuits (Avolio, 2020; Cohen & Garcia, 2008). As women in engineering have lower levels of belonging than men (Wilson & VanAntwerp, 2021), it is plausible they are less likely to be retained in engineering. Therefore, it is important to better understand what factors contribute to this lowered sense of belonging and what consequences this has.

The importance of belonging for academic persistence is well documented, but *how* those feelings of belonging develop naturalistically is still an open question. We hypothesize that, in engineering and other STEM disciplines that employ project-based learning approaches, having the opportunity to engage in technical participation early in one’s academic career (i.e., in the first year of college) may be one route to fostering a sense of belonging. Further, an increase in sense of belonging stemming from early technical participation may facilitate longer-term persistence in these activities and disciplines.

Unfortunately, the opportunity to engage in technical participation is not equitable in many group project teams. Across many studies of small group interactions, men have been shown to talk more and more assertively, to talk longer, and to be more likely to emerge as leaders compared to women (Leaper & Ayres, 2007; Lewis et al., 2019; Rosser, 1998; Sekaquaptewa & Lewis, 2018). This gender difference in participation in small group interaction emerges more strongly among undergraduates (compared to older adults) and among those less familiar with one another (Leaper & Ayres, 2007). Men’s dominance is more pronounced in mixed gender groups (as individual men tend to dominate more in the presence of women than with other men), and when the group is engaged in men-typed tasks (Okudan & Mohammed, 2006). Men-typed tasks are characterized by being non-personal and instrumental (focused on accomplishing a goal), as compared to women-typed tasks, characterized as interpersonal and relational (Eagly & Karau, 1991). Of note, the characteristics promoting gender differences in small group interactions identified in this research (i.e., mixed-gender groups of undergraduates working on men-typed tasks) are also characteristic of undergraduate engineering and other STEM group project teams. These characteristics may partially explain why women often report not feeling included in engineering (Ayre et al., 2013; Faulkner, 2009; Good et al., 2012), feelings that emerge as early as their first year in college (Cech et al., 2011).

Indeed, a detailed analysis of student behaviors in engineering group project presentations indicated that gender-stereotypic role adoption emerges in undergraduate engineering student groups^1^. Specifically, Meadows and Sekaquaptewa (2011) found a gender gap in roles adopted on engineering student teams, reflecting stereotypes of men as engineering experts and women as supporters. In an analysis of video footage of the final group project presentations of over 1000 first year students on mixed-gender teams, men were disproportionately more likely to present technical content than women, to speak longer than expected and longer than women, and to field more audience questions than women. Follow-up surveys and focus groups conducted with subsets of these students revealed that adopting more active technical roles was seen as more desirable and important (Meadows & Sekaquaptewa, 2013). This in turn predicted greater self-perceived learning about engineering among women, with women being the least likely to adopt these roles.

While very informative, those prior studies leave some critical questions unanswered. First, students reported greater self-perceived learning if they presented technical content rather than non-technical content (Meadows & Sekaquaptewa, 2011, 2013). From those findings, it is unclear whether presenting technical content may have long-term benefits beyond self-perceived learning, benefits such as increasing the likelihood of remaining in the field, including graduating with an engineering degree, and intentions to pursue graduate study or a career in engineering. Answering that question requires a longitudinal approach. Second, how does any effect of technical participation on educational outcomes compare to other predictors, such as level of preparation? Is it the case that if students enter STEM well prepared (e.g., with high ACT math scores) they will thrive, or do the behaviors that emerge in the STEM context matter above and beyond pre-college preparation (Dasgupta et al., 2015; Nisbet et al., 2015; Ramsey et al., 2013)? Answering these questions is important for our theoretical understanding of the role technical participation plays in STEM outcomes, and our practical understanding of how to improve STEM education.

## 2. Current Study

Taken together, prior research suggests but has not yet explicitly tested the possibility that men’s and women’s differential participation in first year engineering project teams influences their sense of belonging in the field, or the possibility that changes in feelings of belonging resulting from differential participation have long-term consequences. The current study addresses this gap in the literature, testing three core predictions. We predict that, consistent with prior research (Leaper & Ayres, 2007; Meadows & Sekaquaptewa, 2011), men will be more likely than women to engage in technical participation in first year engineering project teams. In line with previous research showing the importance of belonging in students’ persistence in the field (Cech et al., 2011), we also predict sense of belonging will relate to long-term outcomes (engineering retention, intentions to pursue graduate education, intentions to pursue a career in engineering). More specifically, we predict that early engagement in technical participation will be associated with an increased sense of belonging in engineering, and, in turn, having a greater sense of belonging will be associated with a higher likelihood of being retained in engineering (i.e., completing an engineering degree) and intentions to either pursue a career in engineering or post-graduate education in engineering. That is, we predict that the effect of first year technical participation on retention and intentions will be mediated by sense of belonging in engineering. We also expect these effects to be moderated by gender, such that women will have lower levels of technical participation than men, and therefore a reduced sense of belonging and in turn reduced retention and intentions to remain in the field.

We are also interested in exploring whether other constructs that the prior literature has found to influence gender gaps in STEM mediate the relationship between gender differences in technical participation and long-term outcomes. Therefore, for exploratory purposes, we include several exploratory mediator measures in our survey and assess whether they are associated with gender and long-term outcomes to inform our analysis plan. These constructs were drawn from previous studies in the broader behavioral science of the STEM education literature and included identification with engineering, endorsement of stereotypes, concerns about being stereotyped, and future plans in engineering. Prior research found that women who are highly identified with their gender and those who endorse stereotypes about women’s mathematical abilities tend to perform worse than women with low gender identification and low stereotype endorsement (Schmader, 2002; Schmader et al., 2004). Therefore, we also assess the extent to which endorsement of stereotypes of gender in engineering and the extent to which participants identify with engineering plays a role in shaping long-term outcomes. Moreover, the extent to which women endorse gender stereotypes predicts intentions to continue to study a STEM subject (Schmader et al., 2004), so we investigate whether intentions to continue studying a STEM subject relate to actual behaviors (i.e., retention). Having a welcoming STEM environment has also been shown to reduce the extent to which women have concerns about being stereotyped (Ramsey et al., 2013), and thus we examine the extent to which gender differences in technical participation influence concerns about being stereotyped.

## 3. Materials and Methods

### 3.1. Participants

Participants in this study were 589^2^ undergraduate students (434 men; 153 women; 2 unreported) recruited from the college of engineering at a large Midwestern U.S. university. Of these students, 389 reported their race/ethnicity (67% White, 21% Asian, 6% Hispanic, 3% Mixed Race, 3% Black/African American). Students were recruited from a first year introductory engineering course in 2013-14 and completed a survey in their first year. A sub-sample of 122 of these students completed a second survey in their final year in 2017.

### 3.2. First Year Procedure

***First year technical participation.*** We worked with the college of engineering to recruit students who were enrolled in a first year introduction to engineering course to participate in this study. As part of the curriculum, they completed a semester-long team design project and presented their final designs in class. One advantage of collaborating with the college of engineering is that the college videotapes and archives students’ first year presentations, which enabled us to have objective measures of students’ technical participation—at least to the extent that what they present in their final presentation reflects their engagement in technical participation throughout the course. To assess participation, we used a previously validated behavioral coding scheme (Meadows & Sekaquaptewa, 2011, 2013) to code the videos for the amount of technical and non-technical information that students presented during their presentation. This coding scheme was developed with the college of engineering’s director of first year programs to ensure that it was an ecologically valid measure of technical participation in those first year engineering courses. Two independent raters (identifying as a man and a woman) were trained to score each group’s presentation on the content presented by each student (technical vs. non-technical). Technical aspects of the presentation included the detailed description of the design solution, technical specifications, calculations, and/or analyses. Non-technical aspects included the title slide or concluding slide, introduction, implications, and/or summary. Because our theoretical predictions are focused on the longitudinal effects of technical participation, we will only present those findings in this paper; we do however present the effects of non-technical participation in the online Supplementary Materials for interested readers. We operationalize technical participation as whether the student presented at least one technical content slide or slides in their group’s final presentation (0 = they did not present a technical content slide, 1 = they did present a technical content slide). Raters were easily able to identify which slides contained technical content as evidenced by the high level of inter-rater agreement on how many slides contained technical content (*r* = 0.76, *p* < 0.001). Therefore, we used the responses of the rater with the most conservative estimate of technical content presented.

***First year survey materials.*** Immediately after delivering their presentation, we gave students a short survey about their experiences both in the group project and in engineering more broadly. In this survey, students rated their level of agreement with survey items on scales of 1 (strongly disagree) to 7 (strongly agree). We measured a variety of constructs shown in prior laboratory studies to predict performance and persistence on STEM-related tasks. Our goal in measuring all of these constructs was to determine which of them were associated with long-term outcomes like retention. These constructs included: sense of belonging (Cheryan et al., 2009), identification with engineering (adapted from Schmader, 2002), endorsement of engineering stereotypes (adapted from Schmader et al., 2004), concerns about stereotyping (Ramsey et al., 2013), and future plans in engineering (Schmader et al., 2004). We found that only sense of belonging was related to *both* gender and retention (see Table 1). Therefore, in the interest of making a complex set of results easier to process, the main text will only focus on sense of belonging. However, all measured constructs are included in the online Supplementary Materials for interested readers.

*First year sense of belonging in engineering* was assessed using four items (α = 0.81). Two were adapted from Cheryan et al. (2009): “I feel like I really belong in the field of engineering; I feel accepted by other students in the field of engineering”. We created two additional items: “I feel accepted by the instructors in the field of engineering; I regret choosing the field of engineering (reverse scored)”. Higher numbers indicate a greater sense of belonging in engineering.

In addition, participants reported demographic information including gender.

### 3.3. Final Year Follow-Up Procedure

Three years later, we sent an invitation to participate in an online survey to all students classified as graduating seniors (final year students) who were enrolled in their final engineering capstone design course (*n* = 1156). These capstone design courses are highly technical in nature and represent a culminating and comprehensive experience for students within the engineering majors. Three hundred thirty-one students completed this survey and were compensated with an entry into a lottery to win one of ten one-hundred-dollar prizes. Of the 331 students that responded to the final survey, 122 had been in our initial first year sample. These 122 students are used for our longitudinal survey analyses.

***Final year survey materials.*** In the final year survey, we asked students about their experiences in their final capstone design project group and in engineering more broadly. We measured the same general constructs as in the first year survey in order to assess changes over time, and their consequences.

*Self-reported final year capstone project participation* was assessed with four items (α = 0.59): “While working on my final design project, I considered myself to be an organizer or leader; While working on my final design project, I generally kept quiet and listened to the others on my team (reverse coded); When my team made a group presentation, I did a lot of the talking; I didn’t have much to contribute when my team made a group presentation (reverse coded)”. Higher numbers indicate a higher level of capstone project participation. Thus, in contrast to first year technical participation (which was observed), final year participation was self-reported and was based on perceptions of active participation in a highly technical course project as a whole, rather than within the final presentation only.

*Final year sense of belonging in engineering* was assessed with the four items from the first year survey and one additional item (α = 0.87): “I feel like I have a lot in common with other students in the field of engineering”. Again, higher numbers indicate a greater sense of belonging in engineering.

*Final year intentions to pursue graduate education in engineering* was assessed by asking students “How likely is it that you will pursue a graduate study related to engineering?” This item was modified based on a similar item used by Schmader et al. (2004).

*Final year intentions to pursue a career in engineering* was assessed by asking students “How likely is it that your eventual career after graduation will directly pertain to engineering?” This item was identical to an item used by Jones et al. (2010).

### 3.4. Administrative Data Collection

At the end of the semester after the final year survey was completed, we retrieved administrative data (including mathematics ACT score, current/final major, whether they had completed a degree or were still enrolled) from students’ records held by the university registrar. These data allowed us to assess retention (0 = graduated with a degree in a major other than engineering or still enrolled in a major other than engineering, 1 = graduated with a degree in engineering or still enrolled in an engineering major).

Finally, respondents’ data from these four sources—coding of classroom presentation videos, two surveys, and registrar data were then matched and merged into a single database for a comprehensive longitudinal analysis.

## 4. Results

### 4.1. Preliminary Analysis

We first examined bivariate correlations among the core variables of interest, gender and math ACT score—a variable posited to predict success in engineering. This allowed us to determine if these variables influence patterns of results and thus should be controlled for or entered as factors in our main analyses. As illustrated in Table 2, Math ACT was not significantly related to our variables of interest but was approaching significance for first year sense of belonging (r = 0.10, *p* = 0.070). Given these findings and previous research showing that Math ACT predicts intentions to pursue STEM education (D. I. Walton, 2023), we include Math ACT score as a covariate in our main analyses. We report analyses without Math ACT being controlled for in the Supplementary Materials and found no substantial differences.

Second, we checked to see if there were any substantive sample differences (gender, ethnic group, math ACT scores, and team gender composition) and our independent variable differences (first year technical participation and first year sense of belonging) between the 122 students who completed the final year survey and the rest of the sample that did not (see Table 3) using independent samples *t*-tests for continuous variables and chi-square for categorical variables. This allows us to determine if the sample used for our longitudinal analysis is biased in any systematic ways. We could not find any significant predictor of participation in the final survey, and thus we proceeded with our longitudinal analyses.

***Gender Differences in First Year Technical Participation.*** To test our first hypothesis that men engage in technical participation more than women in first year engineering project teams, we conducted a logistic regression with technical participation (0 = did not present technical information, 1 = presented technical information) as the dependent measure, gender as the predictor, and math ACT score as a covariate. We conducted this analysis on mixed-gender teams (70% of teams for which team composition data were collected, *n* = 204) only, because it would be impossible for women to engage in technical participation in all-men teams or for men to engage in technical participation in all-women teams. We found a main effect of gender on technical participation (*b* = −0.33, Wald = 5.10, *p* = 0.024, *Exp* (*B*) = 0.72, 95% CI [0.54, 0.96]), whereby, in mixed-gender teams, men were more likely to engage in technical participation than women. This result indicates that gender differences in technical participation emerge in first year engineering project teams, replicating prior research, albeit with a different operationalization (e.g., Meadows & Sekaquaptewa, 2011; Okudan & Mohammed, 2006).

To examine the consequences of this technical participation, we return to using the full sample (*n* = 388 as Math ACT data were missing for multiple participants) to test the remaining predicted relations. This sample included a range of team gender compositions (*n* = 293 due to missing data): 44.7% men-dominated, 25.3% all men, 14.3% women-dominated, 10.6% equal gender distribution, 5.1% all women. There could be differences in technical participation depending on the gender composition, as being the only woman in a team can have detrimental effects for the performance of women in men-dominated teams (Thompson & Sekaquaptewa, 2002), and women participate more in women-majority groups than mixed-gender groups or men-dominated groups (Dasgupta et al., 2015). Therefore, we assessed whether team gender composition affected technical participation. We found technical participation did not significantly differ depending on the team gender composition (*b* = −0.13, Wald = 1.56, *p* = 0.212, *Exp* (*B*) = 0.88, 95% CI [0.71, 1.08]).

***First Year Sense of Belonging in Engineering***. To test the hypothesis that technical participation is associated with sense of belonging in the first year, we conducted a linear regression with first year sense of belonging as the dependent measure and first year technical participation, gender, math ACT scores, and the interaction between gender and first year technical participation (predictors were mean-centered prior to the computation of interaction terms in all regression analyses presented). This allows us to test (1) whether there is an association between first year technical participation and first year sense of belonging and (2) whether that association is moderated by gender (see Table 4). The linear regression revealed a main effect of first year technical participation and gender on first year sense of belonging (both approaching significance), whereby men and those who engaged in technical participation had a higher sense of belonging than women and those who did not engage in technical participation. We did not find main effects of math ACT, and the main effect of technical participation on belonging was not moderated by gender. Our next question was whether the increased sense of belonging that comes from engaging in technical participation in the first year mattered for student retention.

***Engineering Retention***. To test the hypothesis that first year sense of belonging predicts engineering retention, we conducted a binary logistic regression with retention (1 = student retained, 0 = not retained) as the dependent measure and first year technical participation, first year sense of belonging, gender, math ACT score, and the interactions between technical participation and belonging with gender (see Table 5). This allowed us to test (1) whether there is a relationship between first year sense of belonging and retention and (2) whether such a relationship is moderated by gender. The logistic regression revealed a main effect of sense of belonging on retention, whereby those with a higher sense of belonging in their first year were more likely to be retained in engineering in their final year. There were no other significant predictors of retention, nor were the effects of first year belonging moderated by gender. These findings suggest that a student’s sense of belonging during their first year in engineering predicts their likelihood of staying enrolled in engineering independent of their gender. In the Supplementary Materials, we also report a model that includes final year sense of belonging; it is important to note that, with this model, we do not find any significant effects. However, this model was substantially underpowered (*n* = 70) due to missing data for final year sense of belonging and Math ACT.

**Figure 1:**
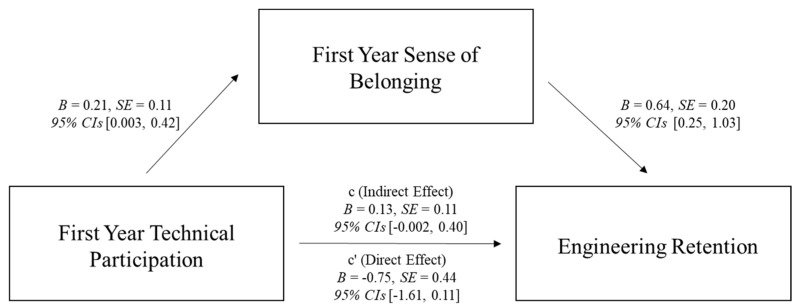
Mediation model showing the relationship between first year technical participation and retention was not mediated via first year sense of belonging.

***Mediation Model Between First Year Technical Participation and Retention Mediated* via *First Year Sense of Belonging***. Next, we tested a simple mediation model looking at whether the relationship between first year technical participation and retention was mediated via first year sense of belonging using PROCESS macro version 4 (Hayes, 2013) with 5000 bootstrapped samples (see Figure 1 for full statistics), when controlling for Math ACT. No technical participation was the reference category. Although each path of the model was in the predicted direction, the overall indirect effect was non-significant. This could be due to a lack of statistical power due to only having retention and Math ACT data for 328 (of the total 589) participants. We ran a post hoc power analysis using a Monte-Carlo power analysis calculator (Schoemann et al., 2017), which indicated we only had 31% power to be able to detect this effect. However, it is also plausible that the lack of significance for the overall model means this effect is not robust or does not exist; future research should investigate this relationship further with larger longitudinal samples.

***Post-Graduation Intentions***. To test the hypothesis that sense of belonging predicts post-graduation intentions, we conducted two linear regressions with final year intentions to pursue engineering graduate education or final year intentions to pursue an engineering career as the dependent measure and first year technical participation, first year sense of belonging, final year sense of belonging, gender, math ACT score, and the interactions with gender as the predictors. This allowed us to test (1) whether there is a relationship between first year sense of belonging and two types of post-graduation intentions and (2) whether such a relationship is moderated by gender.

The first regression revealed a main effect of both first and final year sense of belonging on final year intentions to pursue engineering graduate education whereby those with a higher sense of belonging in their first or final year were more likely to intend to pursue engineering graduate education in their final year (see Table 6). There were no other significant predictors of intentions to pursue engineering graduate education nor were the effects of sense of belonging moderated by the gender. These findings suggest that a student’s sense of belonging in engineering predicts their likelihood of pursuing graduate education in engineering regardless of their gender.

The second regression did not reveal a main effect of first year sense of belonging on final year intentions to pursue an engineering career (see Table 7). However, final year sense of belonging did predict final year intentions to pursue an engineering career, and this effect is moderated by gender, such that the relationship between final year sense of belonging and post-graduate career intentions is stronger for women than men. There were no other significant predictors of retention. These findings suggest that although a student’s sense of belonging during their first year in engineering does not predict the distal outcome of their likelihood of pursuing a career in engineering, their final year sense of belonging does predict the more proximal outcome of their likelihood of pursuing a career in engineering, regardless of their gender.

**Figure 2:**
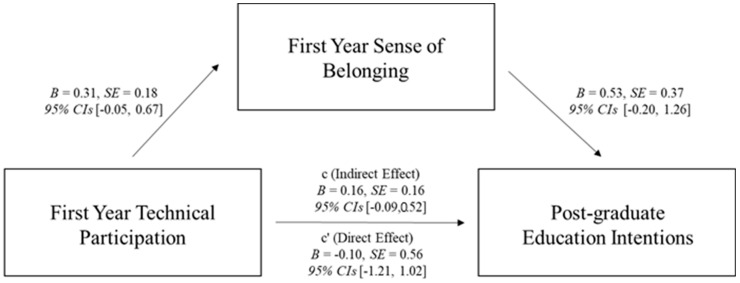
Mediation model showing the relationship between first year technical participation and post-graduate education intentions was not mediated via first year sense of belonging.

**Figure 3:**
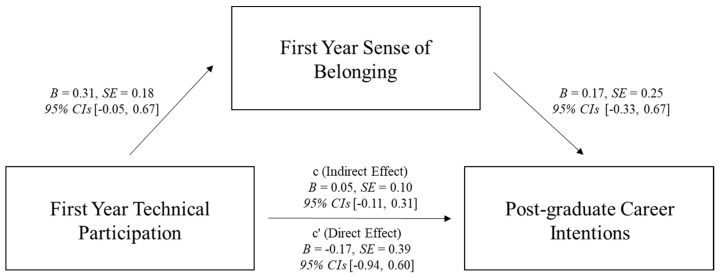
Mediation model showing the relationship between first year technical participation and post-graduate career intentions was not mediated via first year sense of belonging.

We also tested two simple mediation models investigating whether the relationship between first year technical participation and post-graduate intentions (education and career) was mediated via first year sense of belonging using PROCESS macro version 4.2 (Hayes, 2013) with 5000 bootstrapped samples (see Figure 2 and Figure 3 for full statistics), when controlling for Math ACT. No technical participation was the reference category. Although each path of the model was in the predicted direction, the overall indirect effect was non-significant. We suspect that the lack of significance was due to a lack of statistical power due to only having post-graduate intentions and Math ACT data for 72 (of the total 589) participants. We ran a post hoc power analysis using a Monte-Carlo power analysis calculator (Schoemann et al., 2017), which indicated we only had 3% and 2% power for the post-graduate education and career models, respectively. However, it is also plausible that the lack of significance for the overall model means this effect is not robust or does not exist; future research should investigate this relationship further with larger longitudinal samples.

### 4.2. General Discussion

Overall, the results of this study suggest that students’ sense of belonging in engineering is a critical factor underlying the effects of first year technical participation in engineering project groups on long-term outcomes. This research provides some insights into potential sources of the gender differences in retention that plague engineering and other STEM fields (e.g., Cheryan et al., 2017; Hill & Corbett, 2015; Lord et al., 2011). The longitudinal analyses revealed that technical participation is related to students’ sense of belonging in engineering and ultimately their retention in the field—both in the undergraduate major and in terms of their post-graduation intentions. As women are less likely than men to engage in technical participation, they are less likely to experience these long-term benefits of technical participation. These results have important implications for research on, and teaching practices in, group dynamics, engineering, and STEM education more broadly.

### 4.3. Implications for Theory

This work contributes to a growing body of literature on sense of belonging—a literature demonstrating that the extent to which people feel like they belong has vast implications for motivation and persistence (G. M. Walton et al., 2023; G. M. Walton & Cohen, 2011). Research on sense of belonging has documented that having a greater sense of belonging fosters the development of intrinsic motivation (e.g., Anderman & Anderman, 1999) and increases students’ intentions to persist (Marra et al., 2013). While brief interventions have been developed to foster a sense of belonging (G. M. Walton & Cohen, 2011), the literature has been largely silent on how belonging is fostered naturalistically over time. The current research addresses this gap in knowledge, demonstrating that, at least in the context of engineering learning environments, the roles students adopt and the behaviors they engage in may affect their sense of belonging. Students who engaged in technical participation early in their first year had a higher sense of belonging, and this was associated with positive downstream outcomes—in particular, outcomes related to persistence in engineering and future career intentions.

The present research also contributes to the literature on engineering education. Many engineering schools and colleges have adopted project-based learning techniques in an effort to increase student retention. Prior research documented that these approaches increase the development of technical and professional skills (e.g., Severiens & Schmidt, 2009) and overall academic performance (Haidet et al., 2014). Our research demonstrates that project-based learning can also increase retention in engineering to the extent that students engage in technical participation in their project groups. Engaging in technical participation was associated with greater sense of belonging in the field, which in turn was associated with a higher likelihood that students persist and graduate with an engineering degree, and stronger intentions to pursue engineering related advanced degrees and careers. Interestingly, both first and final year sense of belonging were associated with intentions to pursue engineering education, but only final year sense of belonging was related to intentions to pursue a career in engineering. This highlights the importance of ensuring students feel a sense of belonging throughout their academic trajectory and dovetails with previous work. During a student’s first year of college education, the need to feel a sense of belonging is especially strong as they are transitioning to and trying to integrate themselves into a new environment (Araújo et al., 2014; Maunder, 2018). However, a sense of belonging remains important throughout college education as students continue to transition to new stages with new expectations and challenges (Maunder, 2018).

The current research also highlights the need for engineering (and perhaps other STEM) educators to consider the role that gender and gender stereotypes may play in shaping dynamics and role adoption in student groups (for a longer discussion, see Lewis & Sekaquaptewa, 2016). As documented in this study, opportunities to engage in technical participation, which seem to be important for fostering a sense of belonging and retention, were not equal in student project groups. Perhaps due to stereotypes of women as less technically competent (Meadows & Sekaquaptewa, 2011), women were less likely to adopt technical roles in mixed-gender teams. This places women at a disadvantage in terms of not only fostering the sense of belonging necessary to persist in the field, but perhaps also in gaining the technical preparation required to excel in their careers.

Of interest, gender differences in types of roles adopted in teams (technical vs. organizational) may be detrimental for men as well as women. For men, the lack of experience in developing professional skills can negatively impact their preparation for the engineering workforce. In the Transforming Undergraduate Education in Engineering Phase I Workshop report (ASEE, 2013), participants from industry and academia point to good communication skills and the ability to collaborate within a diverse workforce as two of several key qualities of a well-prepared graduate. To the extent that they do not engage in organizational roles that would foster their development in these areas, men students may be disadvantaged when these roles go primarily to women students.

Interestingly, we did not find that first year technical participation was correlated with final year technical participation. In the first year, technical participation was measured by observing the students’ final presentations, whereas, in the final year, participants self-reported their technical participation. This difference in measurement (observed vs. self-reported) potentially accounts for why these variables were not correlated. Future research should investigate and consider this when deciding how to measure technical participation.

### 4.4. Implications for Practice

From a practical perspective, the current research highlights critical factors that contribute to the problem of attrition among STEM students (National Science Board, 2014; President’s Council of Advisors on Science and Technology, 2012). High attrition undermines the goal of increasing the size of the STEM workforce (President’s Council of Advisors on Science and Technology, 2012; U.S. Department of Education, 2022) and retention gaps for women and under-represented minorities undermine the goal of having a high-quality STEM workforce (Page, 2007, 2019). The current data suggest that, at least in the context of engineering, first year experiences set students on a long-term career path, but whether or not they stay on this path and persist in the field depends on their sense of belonging—the extent to which they believe people like them are valued and accepted in their field. This sense of belonging is again associated with whether they engage in technical participation early on in the field, which seems to be influenced by students’ gender and perhaps stereotypes about gender roles (Meadows & Sekaquaptewa, 2011). These results imply that, to address issues of attrition and diversity in STEM, it is important for STEM educators and practitioners to provide equal opportunities for people to engage with technical aspects of their field, and address stereotypes and biases that may undermine these opportunities (Devine et al., 2012). This should increase a sense of belonging and in turn long-term retention.

In the context of teaching in a project-based learning environment, faculty can influence important outcomes for students. By shifting norms and expectations and actively promoting an egalitarian environment, faculty can play a significant role in creating a learning environment in which racism and sexism (explicit or implicit) are neither expected nor tolerated (Bennett & Sekaquaptewa, 2014). Faculty may also structure courses and assignments in ways that supports equity in participation and learning for all students. Meadows et al. (2015) provided several instructional strategies, including establishing role models for appropriate collaboration, addressing both process and product in scaffolding and evaluating group assignments, and establishing peer interaction patterns that support collaboration and reduce anxiety.

It is worth noting that while first year sense of belonging predicts intentions to pursue post-graduation engineering education, it does not predict intentions to pursue a career in engineering. However, final year sense of belonging does predict career intentions. Moreover, sense of belonging in the first year and final year were weakly correlated, suggesting that sense of belonging might change over time. Therefore, it is important that educators focus on developing a sense of belonging throughout the education process, for example, through mentorship (Apriceno et al., 2020). Indeed, previous research has highlighted the benefits of women mentors and having a range of mentors at different academic levels for women’s sense of belonging (Dennehy & Dasgupta, 2017; Du et al., 2023).

### 4.5. Limitations and Future Directions

Although the current study provides important information about factors that influence retention in engineering, there are, of course, limitations to our findings. First, although we found that first year technical participation predicts first year sense of belonging, which in turn predicts engineering retention, our mediation analysis was not significant potentially due to the longitudinal analysis being underpowered (31% power) due to attrition. Although the limited sample size for longitudinal analysis is a weakness, having *some* longitudinal information about how these processes unfold for real students in an ecologically valid context (i.e., as they navigated their entire academic journey) does provide important insights for the field as well as for educational practitioners. Future researchers can build on these insights by perhaps forming multi-site consortia that can study these processes on a larger scale (see Moshontz et al., 2018).

Second, technical participation—our primary predictor in the longitudinal analyses—was based on brief final presentations students gave during their first year in college. Those presentations were snapshots of students’ experiences and thus do not capture the nuances of dynamics that unfolded throughout the semester that likely influenced the final presentations that we observed. Future research should address this limitation by observing students’ behaviors multiple times throughout the semester, though it is worth noting that a large body of research on “snap judgments” demonstrates that even small snapshots like we observed can be predictive of long-term outcomes (Rule & Ambady, 2011a, 2011b).

Third, the final survey that provided the data about students’ long-term career intentions contained important confounds—it consisted of students who (a) remained enrolled in the college of engineering and (b) opted to complete a voluntary survey that was mass-emailed to them. We did not collect data from students who dropped out of the college, nor did the college of engineering with whom we worked. Because of this, we may be missing additional information that could provide greater insights into the reasons students leave (and stay), which could be very important for designing interventions focused on increasing retention. Future research in this vein should assess the attitudes of students who leave STEM fields early and students at the time of graduation. Institutions can aid in these efforts by implementing organization-wide exit surveys for all who leave the institution. It is also important to note that another sample limitation is our sample was predominately white, and these effects may be amplified for women from marginalized racial backgrounds due to the “double jeopardy” they experience. This is an important area for future investigation.

Fourth, as discussed in the method section, we measured a variety of constructs shown in prior laboratory studies to predict performance and persistence on STEM-related tasks (e.g., belonging, identification with engineering, endorsement of stereotypes, concerns about stereotyping). We performed these measurements because our reading of the literature suggested that any one of these (or multiple of these) could have been plausible alternative mechanisms that explain differences in long-term outcomes like retention. Of the constructs measured, however, the only one that was related to gender and long-term retention was sense of belonging. Future meta-analytic research should investigate which mechanisms are most important for different types of STEM outcomes—in other words, it should explore important sources of heterogeneity (Bryan et al., 2021).

## 5. Conclusions

Despite its limitations, the present study provides novel insights into the longitudinal process by which the first year undergraduate group experience influences long-term career trajectories in STEM. Having the opportunity to demonstrate competence by engaging in the technical aspects of STEM disciplines fosters a sense of belonging for students that can influence their ultimate career path. These results suggest that a potentially promising way to increase the quantity and quality of the STEM field is to increase students’ technical engagement in an equitable way; that is, to structure our learning environments in ways that give all of our students the opportunity to achieve their full potential.

## Figures and Tables

**Table 1:** Bivariate correlations among variables which previous research has found relate to long-term outcomes.

Variable	*n*	1	2	3	4	5	6	7
1. Gender	587	-						
2. First year sense of belonging	589	−0.10 *	-					
3. First year identification with engineering	588	−0.04	0.65 ***	-				
4. First year endorsement of engineering stereotypes	585	−0.19 ***	−0.08 *	−0.06	-			
5. First year concerns about stereotyping	581	0.39 ***	−0.17 ***	−0.04	0.05	-		
6. First year future plans in engineering	583	0.01	0.63 ***	0.55 ***	−0.06	−0.05	-	
7. Engineering retention	565	<0.01	0.23 ***	0.20 ***	0.06	0.07	0.28 ***	-

Note. Descriptive statistics for each variable: Gender (73.9% men, 26.1% women), first year sense of belonging (range: 1.75–7.00, *M* = 5.76, *SD* = 0.98), first year identification with engineering (range: 1.00–7.00, *M* = 5.00, *SD* = 1.55), first year endorsement of engineering stereotypes (range: 1.00–7.00, *M* = 2.43, *SD* = 1.48), first year concerns about stereotyping (range: 1.00–7.00, *M* = 1.92, *SD* = 1.39), first year future plans in engineering (range: 1.00–7.00, *M* = 5.19, *SD* = 1.20), engineering retention (9.0% were not retained, 91.0% were retained). * *p* < 0.05 *** *p* < 0.001.

**Table 2:** Bivariate correlations among key variables in longitudinal analyses.

Variable	*n*	1	2	3	4	5	6	7	8	9
1. Math ACT score	338	-								
2. Gender	587	−0.03	-							
3. First year technical participation	589	0.08	0.08	-						
4. First year sense of belonging	589	0.10	−0.10 *	0.08 *	-					
5. Engineering retention	565	0.08	<0.01	−0.05	0.23 ***	-				
6. Final year technical participation	124	−0.04	0.07	0.16	<0.01	<0.01	-			
7. Final year sense of belonging	122	0.12	−0.04	0.11	0.18 *	−0.04	0.35 ***	-		
8. Final year intentions to pursue engineering graduate education	122	0.12	0.12	0.05	0.21 *	0.03	0.08	0.28 **	-	
9. Final year intentions to pursue engineer career	122	0.09	−0.08	0.09	0.14	−0.11	0.22 *	0.54 ***	0.33 ***	-

Note. Descriptive statistics for each variable: Math ACT Score (Range: 24–36, *M* = 31.41, *SD* = 2.95), gender (73.9% men, 26.1% women), first year technical participation (70.6% did not present tech, 29.4% presented tech), first year sense of belonging (range: 1.75–7.00, *M* = 5.76, *SD* = 0.98), engineering retention (9.0% were not retained, 91.0% were retained), final year technical participation (range: 3.75–5.50, *M* = 4.86, *SD* = 0.52), final year sense of belonging (range: 1.00–7.00, *M* = 5.55, *SD* = 1.23), final year grad intentions (range: 1.00–7.00, *M* = 4.28, *SD* = 2.29) final year career intentions (range: 1.00–7.00, *M* = 5.69, *SD* = 1.74). * *p* < 0.05 ** *p* < 0.01 *** *p* < 0.001.

**Table 3:** Testing differences in demographic variables and independent variables between the first year and final year samples.

	First Year vs. Final Year Sample
*Sample Variables*	
Gender	X^2^ (*n* = 587) = 2.07, *p* = 0.151
Ethnic Group	X^2^ (*n* = 389) = 2.64, *p* = 0.620
Math ACT Scores	*T*_336_ = −1.18, *p* = 0.239
Team Gender Composition	X^2^ (*n* = 377) = 5.53, *p* = 0.237
*Independent Variables*	
First year technical participation	X^2^ (*n* = 589) = 1.33, *p* = 0.249
First year sense of belonging	*t*_587_ = −1.10, *p* = 0.273

**Table 4:** Linear regression testing the predictors of first year sense of belonging.

	b	SE	t	*p*	95% CI
First year technical participation	0.22	0.11	1.95	0.052	[−0.002, 0.45]
Math ACT	0.03	0.02	1.73	0.085	[−0.004, 0.06]
Gender	−0.11	0.06	−1.96	0.051	[−0.22, <0.01]
First year technical participation × Gender	0.03	0.06	0.53	0.594	[−0.08, 0.14]

**Table 5:** Logistic regression testing the predictors of retention.

	b	SE	Wald	*p*	Exp (B)	95% CI of Exp (B)
First year sense of belonging	0.57	0.21	7.12	0.008	1.77	[1.16, 2.70]
First year technical participation	−0.43	0.51	0.69	0.407	0.65	[0.24, 1.79]
Math ACT	0.08	0.07	1.33	0.248	1.08	[0.95, 1.24]
Gender	−0.05	0.27	0.03	0.861	0.95	[0.56, 1.62]
First year technical participation × Gender	0.42	0.26	2.68	0.102	1.52	[0.92, 2.51]
First year sense of belonging × Gender	−0.31	0.22	2.03	0.154	0.74	[0.48, 1.22]

**Table 6:** Linear regression testing the predictors of post-graduate education intentions.

	b	SE	t	*p*	95% CI
First year technical participation	−0.19	0.59	−0.32	0.747	[−1.36, 0.98]
First year sense of belonging	0.95	0.42	2.26	0.027	[0.11, 1.79]
Final year sense of belonging	0.56	0.22	2.49	0.015	[0.11, 1.01]
Math ACT	0.01	0.10	0.09	0.933	[−0.19, 0.20]
Gender	0.38	0.28	1.32	0.191	[−0.19, 0.94]
First year technical participation × Gender	−0.23	0.29	−0.77	0.443	[−0.81, 0.36]
First year sense of belonging × Gender	0.51	0.42	1.22	0.225	[−0.32, 1.35]
Final year sense of belonging × Gender	0.34	0.23	1.50	0.140	[−0.11, 0.79]

**Table 7:** Linear regression testing the predictors of post-graduate career intentions.

	b	SE	t	*p*	95% CI
First year technical participation	−0.02	0.34	−0.05	0.960	[−0.69, 0.66]
First year sense of belonging	0.32	0.24	1.33	0.190	[−0.16, 0.80]
Final year sense of belonging	0.81	0.13	6.30	<0.001	[0.55, 1.07]
Math ACT	<0.01	0.06	0.02	0.987	[−0.11, 0.11]
Gender	−0.17	0.16	−1.04	0.303	[−0.50, 0.16]
First year technical participation × Gender	0.14	0.17	0.83	0.412	[−0.20, 0.48]
First year sense of belonging × Gender	0.29	0.24	1.21	0.232	[−0.19, 0.77]
Final year sense of belonging × Gender	0.26	0.13	2.02	0.047	[0.003, 0.52]

## Data Availability

The data presented in this study are not available due to privacy.

## Notes

1 Students in this course were generally assigned to teams to facilitate meeting opportunities according to which campus they lived on. After sorting that way, faculty were asked to create teams so as not to isolate under-represented individuals, if possible. It was also recommended not to put roommates on the same team if they were both enrolled in the same section of the course. Outside of these recommendations, the teams were formed randomly.

2 These participants were recruited as part of a larger study of undergraduate engineering students (Meadows & Sekaquaptewa, 2011, 2013). The current analyses are based on a longitudinal follow up of those students that occurred three years later.

## Supplementary Materials

The following supporting information can be downloaded at: https://www.mdpi.com/article/10.3390/bs15020140/s1.
