# Supplementary material for: First Year to Future Career: Women’s Engagement in Technical Participation Is Associated with Long-Term Retention
Source: Behav Sci (Basel). 2025 Jan 27;15(2):140. doi: 10.3390/bs15020140 (PMC11851499; doi:10.3390/bs15020140)
Supplement: Supplementary file 1 [file behavsci-15-00140-s001.zip › behavsci-3318524-supplementary.pdf]

Supplemental Materials to – First Year to Future Career

Correlates of Non-Technical Participation

Bivariate correlations of non-technical participation in longitudinal analysis

Variable	First year non-technical participation
1. Math ACT score	-0.01
2. Gender	-0.06
3. First year sense of belonging	0.03
4. Engineering retention	-0.01
5. Final year sense of belonging	0.08
6. Final year intentions to pursue engineering graduate education	0.15
7. Final year intentions to pursue engineer career	0.01

Note. * $p<0.05$ ** $p<0.01$ *** $p<0.001$

First Year Survey Measures

ProjectLeader (α=0.467)

- While working on our group project, I considered myself to be an organizer or leader.
- While working on our group project, I generally kept quiet and listened to the others on my team (R).

PresentationLeader (α=0.340)

- During our group presentation today I did a lot of the talking.
- I didn't have much to contribute to our group presentation today (R).

NoChoiceInRole (α=0.640)

- I believe I had some choice about what role I adopted on the group project (R).
- I adopted the role that I did on the group project because I had to.
- I was pressured by others to take on the role I adopted on the group project.
- I felt like others would disapprove of me if I did not adopt the role that I did on the project.

SatisfactionWithGroupRole

- I am satisfied with the role I played on the group project

SatisfactionWithPresentationRole

- I am satisfied with the role I adopted during the group presentation today.

SatisfactionWithProjectPerformance (α=0.520)

- I feel good about my individual performance on the group project
- I wish I had done a better job on the group project (R).

SatisfactionWithPresentationPerformance (α=0.532)

- I feel good about my individual performance during the group presentation today.
- I wish I had done a better job during the group presentation today (R).

SelfPerceivedLearning_Project (α=0.787)

- I feel like I learned a lot doing this group project.
- I actually didn't get a lot out of doing this group project (R).
- My understanding of the course material was strengthened by doing this group project.

SelfPerceivedLearning_Presentation

- I believe I learned a lot about the material by presenting it to others during our group presentation today.

WouldLearnMoreIfMoreActive

- I think I would have gotten more out of the group project if I had taken a more active role during the group presentation today.

TeacherSupporter

- During group presentations, people sometimes take on an active "teacher" role, in which they primarily impart technical or other critical information to others. Other times people take on a "supporter" role in which they primarily help or support other presenters. In your group

GroupRanking

- Please rank each group member on their level of contribution to the completion of your group project (using 1 for the person who contributed most, 2 for the person who contributed second most, etc.).

SenseofBelonging (α=0.812)

- I feel like I really belong in the field of engineering.
- I feel accepted by other students in the field of engineering.
- I feel accepted by the instructors in the field of engineering.
- I regret choosing the field of engineering (R).

IdentificationWithEngineering

- Being a student in the field of engineering is an important part of who I am.

MyGenderBelongs (α=0.774)

- People of my gender are welcome in the field of engineering.
- I feel like people treat me negatively in the field of engineering because of my gender (R).

MyRaceBelongs (α=0.638)

- People of my race/ethnicity are welcome in the field of engineering.
- I feel like people treat me negatively in the field of engineering because of my race/ethnicity (R).

StereotypeEndorsement (α=0.856)

- It is possible that men have more engineering ability than women.
- In general, men may be better than women at engineering.
- I don't think that there are any real gender differences in engineering ability (R)

StereotypeThreatConcern (α=0.899)

- I am concerned that people will judge my gender as a whole based on my performance on this group project.
- I am concerned that people will think that my gender group as a whole has less ability if I do not do well on this project.

IntentionstoPersistInEngineering (α=0.730)

- How likely is it that you will pursue a graduate study related to engineering?
- How likely is it that your eventual career after graduation will directly pertain to engineering?
- How often do you think about changing your major? (R)
- How likely is it that you will change your major? (R)

TraditionalGenderRoleEndorsement (α=0.739)

- Swearing and obscenity are more repulsive in the speech of a woman than a man.
- Under modern economic conditions, with women active outside the home, men should share in household tasks such as washing dishes and doing laundry. (R)
- It is insulting to women to have the "obey" clause still in the marriage service. (R)
- A woman should be as free as a man to propose marriage (R).
- Women should worry less about their rights and more about becoming good wives and mothers.
- Women earning as much as their dates should bear equally the expense when they go out together (R).
- Women should assume their rightful place in business and all the professions along with men (R).
- A woman should not expect to go to exactly the same places or to have quite the same freedom of action as a man.
- Sons in a family should be given more encouragement to go to college than daughters.
- It is ridiculous for a woman to run a locomotive and for a man to sew.
- In general, the father should have greater authority than the mother in raising the children.
- The intellectual leadership of a community should be largely in the hands of men.
- Economic and social freedom is worth far more to women than acceptance of the ideal of femininity, which has been set up by men (R).
- There are many jobs in which men should be given preference over women in being hired or promoted.

Final Survey Measures

ProjectLeader (α=0.327)
- While working on my senior design project, I considered myself to be an organizer or leader.
- While working on my senior design, I generally kept quiet and listened to the others on my team (R).

PresentationLeader (α=0.405)
- When my team made a group presentation, I did a lot of the talking.
- I didn't have much to contribute when my team made a group presentation (R).

NoChoiceInRole (α=0.656)
- I believe I had some choice about what role I adopted on my senior design project team (R).
- I adopted the role that I did on my senior design project team because I had to.
- I was pressured by others to take on the role I adopted on my senior design project team.
- I felt like others would disapprove of me if I did not adopt the role that I did on my senior design project team.

SatisfactionWithGroupRole
- I am satisfied with the role I played on my senior design project team

SatisfactionWithPresentationRole
- I am satisfied with the role I adopted during my senior design project presentation.

SatisfactionWithProjectPerformance (α=0.682)
- I feel good about my individual performance on my senior design project team
- I wish I had done a better job on my senior design project team (R).

SatisfactionWithPresentationPerformance (α=0.688)
- I feel good about my individual performance during our senior design project presentation.
- I wish I had done a better job during my senior design project presentation (R).

SelfPerceivedLearning_Project (α=0.876)
- I feel like I learned a lot doing my senior design project.
- I actually didn't get a lot out of doing this senior design project (R).
- My understanding of the course material was strengthened by doing this senior design project.

SelfPerceivedLearning_Presentation
- I believe I learned a lot about the material by presenting it to others during my senior design project.

WouldLearnMoreIfMoreActive

- I think I would have gotten more out of the group project if I had taken a more active role during the senior design presentation.

TeacherSupporter

- During group presentations, people sometimes take on an active "teacher" role, in which they primarily impart technical or other critical information to others. Other times people take on a "supporter" role in which they primarily help or support other presenters. In your group presentation today, do you think you took on a teacher or supporter role?

TeamLeader

- Who do you think was the leader in your senior design group? If it was yourself, choose "me." If it was someone else, choose "another person."
  - If another person chosen:
    - My team leader conveyed confidence in my ability to do well on our team project.
    - My team leader encouraged me to ask questions
    - My team leader listened to how I would like to do things.
    - My team leader tried to understand how I saw things
  - If me chosen:
    - I felt I understood the other people on my team.
    - I conveyed confidence in the ability of the others to do well on our team project.
    - I encouraged others on my team to ask questions.
    - I listened to how others on my team would like to do things.
    - I tried to understand how others on my team saw things.

FeltUnderstoodByTeam

- Please respond to the following questions about your perceptions of your other teammates, that is, other people in your group not including the leader.
  - I felt understood by my other teammates.
  - My other teammates conveyed confidence in my ability to do well on our team project.
  - My other teammates encouraged me to ask questions.
  - My other teammates listened to how I would like to do things.
  - My other teammates tried to understand how I saw things.

SenseOfBelonging (α=0.870)

- I feel like I really belong in the field of engineering.
- I feel accepted by other students in the field of engineering.
- I feel accepted by the instructors in the field of engineering.
- I regret choosing the field of engineering (R).
- I feel like I have a lot in common with other students in the field of engineering.

IdentificationWithEngineering

- Being a student in the field of engineering is an important part of who I am.

MyGenderBelongs (α=0.805)

- People of my gender are welcome in the field of engineering.
- I feel like people treat me negatively in the field of engineering because of my gender (R).

MyRaceBelongs (α=0.607)

- People of my race/ethnicity are welcome in the field of engineering.
- I feel like people treat me negatively in the field of engineering because of my race/ethnicity (R).

StereotypeEndorsement (α=0.873)

- It is possible that men have more engineering ability than women.
- In general, men may be better than women at engineering.
- I don't think that there are any real gender differences in engineering ability (R)

StereotypeThreatConcern (α=0.951)

- I am concerned that people will judge my gender as a whole based on my performance on my senior design project.
- I am concerned that people will think that my gender group as a whole has less ability if I do not do well on my senior design project.

EngineeringGradSchoolIntention

- How likely is it that you will pursue a graduate study related to engineering?

EngineeringCareerIntention

- How likely is it that your eventual career after graduation will directly pertain to engineering?

SelfEfficacy (α=0.794)

- When I get new material in engineering, I'm usually sure I will be able to learn it.
- I sometimes doubt my ability to perform well in engineering (R).
- I am good at engineering compared to other people in my major.
- Engineering has always come pretty easy for me.
- Engineering has never been easy for me (R).
- I feel like I have to work harder than other people in engineering to do well (R).

EngineeringIdentification2 (α=0.858)

- Being a student in engineering is an important part of my self-image
- Being a student in engineering is unimportant to my sense of what kind of person I am (R).
- Being a student in engineering has very little to do with how I feel about myself (R).
- Being a student in engineering is an important reflection of who I am.

AttitudesAboutEngineering

- Please rate your feeling towards engineering on the scales below
  - 1 – Good to 7 – Bad
  - 1 – Happy to 7 – Sad
  - 1 – Afraid to 7 – Unafraid
  - 1 – Associated with men to 7 – Associated with women.

Analyses without Math ACT control variable

We ran a chi-square test on the effect of gender on technical participation in mixed-gender teams and found that gender predicts technical participation, X^2 (n=204) = 5.36, p = 0.021, such that women have lower levels of technical participation than men (36.9% vs. 53.3%).

We conducted a linear regression with first year sense of belonging as the dependent measure and first year technical participation, gender, and the interaction between gender and first year technical participation. We found a main effect of first year technical participation and gender on first year sense of belonging, whereby men and those who engaged in technical participation had a higher sense of belonging than those who did not.

Linear regression testing the predictors of first year sense of belonging

	b	SE	t	p	95% CI
First year technical participation	0.24	0.11	2.07	0.039	[0.01, 0.46]
Gender	-0.11	0.06	-1.99	0.047	[-0.23, -0.002]
First year technical participation * Gender	0.03	0.06	0.52	0.605	[-0.08, 0.14]

We conducted a binary logistic regression with retention (1 = student retained, 0 = not retained) as the dependent measure and first year technical participation, first year sense of belonging, gender, and interactions between technical participation and belonging with gender as the predictors. We found a main effect of sense of belonging on retention, whereby those with a higher sense of belonging in their first year were more likely to be retained in engineering.

Logistic regression testing the predictors of retention

	b	SE	Wald	p	Exp (B)	95% CI of Exp (B)
First year sense of belonging	0.58	0.21	7.42	0.006	1.79	[1.18, 2.72]
First year technical participation	-0.38	0.51	0.57	0.451	0.68	[0.25, 1.85]
Gender	-0.05	0.27	0.04	0.850	0.95	[0.56, 1.61]
First year technical participation * Gender	0.41	0.25	2.64	0.104	1.51	[0.92, 2.50]
First year sense of belonging * Gender	-0.33	0.21	2.42	0.120	0.72	[0.47, 1.09]

We conducted two linear regressions with final year intentions to pursue engineering graduate education or final-year intentions to pursue an engineering career as the dependent measure and first year technical participation, first year sense of belonging, final year sense of belonging gender, and the interactions with gender as the predictors. This allowed us to test (1) whether there is a relationship between first year sense of belonging and two types of post-graduation intentions and (2) whether such a relationship is moderated by gender. The first regression revealed a main effect of both first and final year sense of belonging on final year intentions to pursue engineering graduate education whereby those with a higher sense of belonging were more likely to intend to pursue engineering graduate education in their final year. The second regression did not reveal a main effect of first year sense of belonging on final year

intentions to pursue an engineering career. However, final year sense of belonging did predict final year intentions to pursue an engineering career.

Linear regression testing the predictors of post-graduate education intentions

	b	SE	t	p	95% CI
First year technical participation	-0.19	0.58	-0.33	0.743	[-1.35, 0.97]
First year sense of belonging	0.96	0.41	2.34	0.023	[0.14, 1.78]
Final year sense of belonging	0.56	0.22	2.54	0.014	[0.12, 1.00]
Gender	0.38	0.28	1.34	0.184	[-0.18, 0.94]
First year technical participation * Gender	-0.23	0.29	-0.78	0.438	[-0.81, 0.35]
First year sense of belonging * Gender	0.51	0.41	1.24	0.221	[-0.31, 1.33]
Final year sense of belonging * Gender	0.34	0.22	1.54	0.128	[-0.10, 0.78]

Linear regression testing the predictors of post-graduate career intentions

	b	SE	t	p	95% CI
First year technical participation	-0.02	0.33	-0.05	0.959	[-0.68, 0.65]
First year sense of belonging	0.32	0.24	1.36	0.178	[-0.15, 0.80]
Final year sense of belonging	0.81	0.13	6.39	<0.001	[0.56, 1.06]
Gender	-0.17	0.16	-1.05	0.298	[-0.50, 0.15]
First year technical participation * Gender	0.14	0.17	0.83	0.408	[-0.19, 0.47]
First year sense of belonging * Gender	0.29	0.24	1.23	0.224	[-0.18, 0.76]
Final year sense of belonging * Gender	0.26	0.13	2.07	0.043	[0.01, 0.52]

Model on the effect of first and final year sense of belonging on retention

We conducted a binary logistic regression with retention (1 = student retained, 0 = not retained) as the dependent measure and first year technical participation, first year sense of belonging, final year sense of belonging, gender, Math ACT and interactions between technical participation and belonging with gender as the predictors. We found no significant effects.

Logistic regression testing the predictors of retention

	b	SE	Wald	p	Exp (B)	95% CI of Exp (B)
First year sense of belonging	0.87	0.87	1.00	0.317	2.38	[0.44, 13.06]
Final year sense of belonging	-0.79	0.81	0.96	0.328	0.46	[0.09, 2.20]
First year technical participation	-1.15	1.37	0.71	0.399	0.32	[0.02, 4.60]
Math ACT	-0.18	0.24	0.56	0.454	0.84	[0.52, 1.34]
Gender	-0.02	1.07	<0.01	0.986	0.98	[0.12, 7.94]
First year technical participation * Gender	0.16	0.63	0.06	0.803	1.17	[0.34, 4.04]
First year sense of belonging * Gender	-0.87	1.00	0.75	0.385	0.42	[0.06, 2.99]
Final year sense of belonging * Gender	-0.68	0.83	0.67	0.413	0.51	[0.10, 2.57]
